# *Trichinella* T9 in wild bears in Japan: Prevalence, species/genotype identification, and public health implications

**DOI:** 10.1016/j.ijppaw.2023.07.002

**Published:** 2023-07-04

**Authors:** Masaki Murakami, Toshihiro Tokiwa, Hiromu Sugiyama, Mitsuko Shiroyama, Yasuyuki Morishima, Sota Watanabe, Takato Sasamori, Mami Kondo, Tsutomu Mano, Hifumi Tsuruga

**Affiliations:** a Laboratory of Veterinary Parasitology, Nippon Veterinary and Life Science University, Musashino, Tokyo, 180-8602, Japan; b Laboratory of Helminthology, Department of Parasitology, National Institute of Infectious Diseases, Shinjuku, Tokyo, 162-8640, Japan; c School of Life and Environmental Science, Azabu University, Sagamihara, Kanagawa, 252-5201, Japan; d Graduate School of Arts and Science, Iwate University, Morioka, Iwate, 020-8550, Japan; e Agriculture, Forestry and Fisheries Division, Fukaura Town Office, Fukaura, Aomori, 038-2324, Japan; f Nature Conservation Division, Department of Living and Environment, Akita Prefectural Office, Akita, Akita, 010-8570, Japan; g Research Institute of Energy, Environment and Geology, Industrial Technology and Environment Research Department, Hokkaido Research Organization, Sapporo, Hokkaido, 060-0819, Japan

**Keywords:** Bear, Game meat, Public health, *Trichinella*, *Ursus thibetanus japonicus*, *Ursus arctos*

## Abstract

In Japan, the recent series of sporadic outbreaks of human trichinellosis caused by *Trichinella* (Nematoda: Trichocephalida) has occurred owing to the consumption of raw or insufficiently cooked meat from wild bears. However, the infection status and molecular characteristics of *Trichinella* larvae in Japanese wild bears remain poorly understood. This study investigated the prevalence of *Trichinella* spp. in brown bears (*Ursus arctos*) from Hokkaido, and Japanese black bears (*Ursus thibetanus japonicus*) from three prefectures (Aomori, Akita, and Iwate) in northern Japan, between April 2019 and August 2022. *Trichinella* larvae were detected in 2.5% (6/236) of the brown bears and 0.9% (1/117) of the Japanese black bears. Sequence analysis using two genetic loci, the internal transcribed spacer region of nuclear ribosomal DNA and the mitochondrial cytochrome *c* oxidase subunit I gene, revealed that the larvae collected from the seven infected bears were identical to one of the two haplotypes of *Trichinella* T9. The prevalence of *Trichinella* T9 is low but is maintained in bears in the Hokkaido and Iwate prefectures suggesting that undercooked meat from these animals could cause human infection. Thus, continued health education campaigns are needed to raise awareness of the potential risk of trichinellosis among hunters, meat suppliers, consumers, and local governmental health agencies.

## Highlights

• We demonstrated that *Trichinella* is maintained at low levels in wild bears.
• The larvae in brown and Japanese black bears in Japan were identified as *Trichinella* T9.
• Wild bears in Japan may have limited contact with infected animals.
• Consuming bear meat in Japan can transmit *Trichinella* to other animals including humans.

## Graphical abstract

**Figure:**
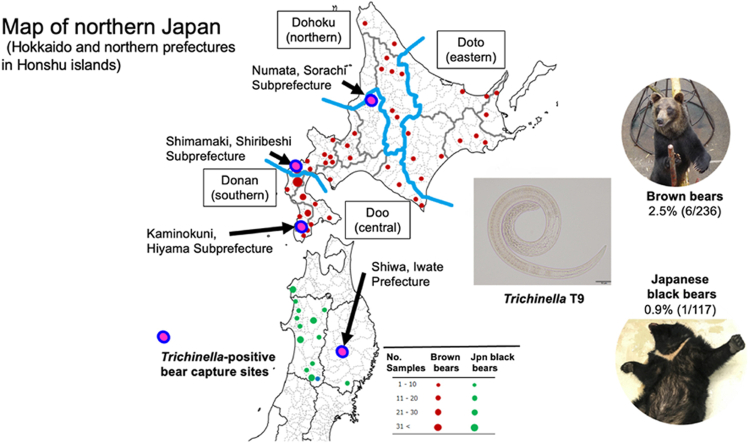

## 1 Introduction

Trichinellosis is a zoonotic disease caused by nematodes of the genus *Trichinella* (Enoplea: Trichocephalida: Trichinellidae). *Trichinella* is transmitted by predation and carrion consumption and circulates between carnivores and omnivores. Therefore, human infection is mainly associated with cultural factors such as consuming raw or undercooked meat from *Trichinella*-infected domestic and wild animals (Pozio and Murrell, 2006).

To date, ten species and three genotypes are recognised in this genus (Zarlenga et al., 2020). Most species/genotypes only infect mammals (*T. spiralis*, *T. britovi*, *T. chanchalensis*, *T. nativa*, *T. nelson*, *T. murrelli*, *T. patagoniensis*, and *Trichinella* T6, T8, and T9). Several other species infect birds (*T. pseudospiralis*) and reptiles (*T. zimbabwensis* and *T. papuae*). A species and a genotype have been recorded from wild animals in Japan. *Trichinella nativa* was detected in red foxes *Vulpes schrencki* (Kanai et al., 2006; Kanai et al., 2007) and *Trichinella* T9 in Japanese black bears *Ursus thibetanus japonicus* (Yamaguchi, 1991; Tominaga et al., 2021), brown bears *Ursus arctos* (Yamaguchi, 1991; Kanai et al., 2007), red foxes *Vulpes schrencki* in Hokkaido (Kanai et al., 2006; Kanai et al., 2007), raccoon dogs *Nyctereutes viverrinus* in Yamagata Prefecture, Honshu (Saito and Yamaguchi, 1985) and in Hokkaido (Kanai et al., 2007), and raccoons *Procyon lotor* in Hokkaido (Kobayashi et al., 2011).

Human trichinellosis is relatively rare in Japan, with only five imported cases (one in 1998, 1999, and 2003, and two in 2009) and 123 domestic cases reported. Of these domestic cases, 119 occurred in six outbreaks (15 in 1974, 12 in 1979, 60 in 1981, 21 in 2016, 3 in 2018, and 8 in 2019), all of which were associated with the consumption of wild bear meat (Yamaguchi, 1991; Tada et al., 2018; Takai, 2022). The etiologic agent of the outbreaks in 1974, 2016, and 2018 was confirmed as *Trichinella* T9, verified by molecular analyses (Nagano et al., 1999; Tada et al., 2018; Morishima et al., 2019). However, studies on the prevalence of *Trichinella* in wild bears are scarce. In brown bears, a prevalence of 3.2% has been reported in Hokkaido (4/126, Kanai et al., 2007). In Japanese black bears, prevalence was 1.2% in Aomori Prefecture (2/161, Yamaguchi, 1991) and 1.4% in Iwate Prefecture (2/144, Tominaga et al., 2021). Therefore, whether the recent human outbreaks, especially those that have occurred after 2016, are due to increased infection rates among wild bears or increasing consumption and preference of wild game, is unclear.

In this study, we investigated the prevalence and molecular characteristics of *Trichinella* larvae in wild bears in Japan. The role of wild bears in the life cycle of *Trichinella* in Japan is also discussed. Furthermore, we examined whether the recent series of human outbreaks were due to changes in the infection status of *Trichinella* in bears or the current popularity of wild meat dishes.

## 2 Materials and methods

### Ethical statement

All samples were collected from wild bears legally hunted for pest control or as game. This study did not involve deliberately killing animals; thus, ethical approval was not deemed necessary.

### 2.1 Sample collection

**Fig. 1:**
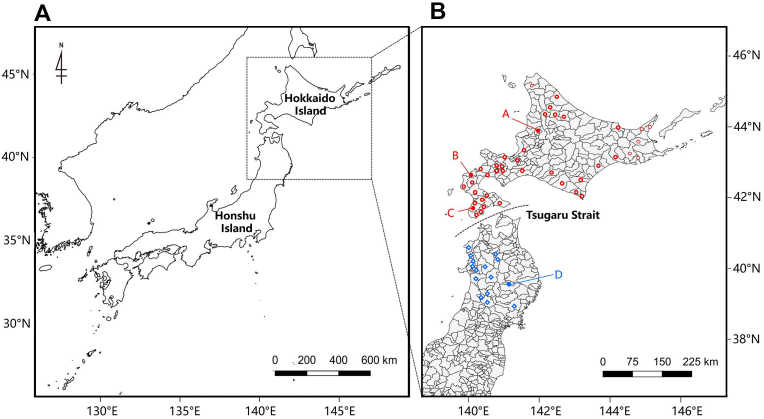
Map of Japan (A) illustrating the bear capture sites in this study (B). The red shapes indicate the capture sites for brown bears (*Ursus arctos*) and blue for Japanese black bears (*Ursus thibetanus japonicus*). The filled shapes indicate the locations of *Trichinella*-positive animals: A, Numata, Sorachi Subprefecture; B, Shimamaki, Shiribeshi Subprefecture; and C, Kaminokuni, Hiyama Subprefecture; D, Shiwa, Iwate Prefecture. (For interpretation of the references to colour in this figure legend, the reader is referred to the Web version of this article.)

Licensed hunters legally hunted 353 wild bears, including 236 brown bears from 13 subprefectures of Hokkaido, and 117 Japanese black bears from Aomori (n = 15), Akita (n = 100), and Iwate (n = 2) prefectures, Japan, for pest control or as game between April 2019 and August 2022. Fig. 1 illustrates the details of the capture sites. Age was determined by counting cementum annual layers of the fourth premolar tooth (Craighead et al., 1970). Tongue samples were collected from all animals by hunters, sent to the co-authors’ offices, and stored frozen at −18 °C until shipping. The samples were then transported while frozen to the National Institute of Infectious Diseases, Tokyo, Japan, twice a year for the detection of *Trichinella* larvae. We requested the shipping companies to accept the samples as clinical specimens such as patient samples from medical settings, and the samples that arrived at this institute were all in good shape, showing no decomposition.

### 2.2 Detection of *Trichinella* larvae

A total of 20 g of the tongue muscle from each bear sample was excised, minced in a food processor, and artificially digested using a PrioCHECK *Trichinella* Alternative Artificial Digestion Kit (Thermo Fisher Scientific, Waltham, MA, USA) according to the manufacturer's protocol. *Trichinella* larvae isolated from each sample in the digestants were mixed thoroughly with physiological saline in a plastic tube up to 50 mL. Subsequently, 5 mL of the solution was transferred to a 9 cm diameter plastic Petri dish. The number of larvae in each dish was counted thrice under a stereoscopic microscope, and the mean number of larvae per gram of tissue (LPG) was calculated. Differences in prevalence between bear species and sexes were separately examined using the chi-squared test.

### 2.3 Molecular identification of *Trichinella* larvae

DNA samples were extracted from a pool of larvae collected from each animal (4–100 larvae/head) using a QIAamp DNA Mini Kit (Qiagen, Hilden, Germany). Two sets of primer pairs (5′- CACCCAGAAGTATACATCC-3′ and 5′-GTAATAATAGGTCTAGGGAGG-3′ for cytochrome *c* oxidase subunit I gene, *cox1,* and 5′-CAATTGAAAACCGGTGAG-3′ and 5′-ATCACTCAACATTAACCG-3′ for the nuclear internal transcribed spacer 2 region, ITS2) were used to identify the *Trichinella* species following the methods described in a previous report (Kanai et al., 2006). The PCR products were visualised using electrophoresis on a 1.5% agarose gel and subjected to direct sequencing at Eurofins Genomics, Inc. (Tokyo, Japan). Multiple sequence alignments were performed using MAFFT ver. 7.505 with the option Q–INS–I (Katoh and Standley, 2016). The sequences obtained were compared to those available in the International Nucleotide Sequence Database at NCBI using the BLASTN program (http://blast.ncbi.nlm.nih.gov/Blast.cgi).

## 3 Results

### 3.1 Prevalence of *Trichinella* larvae in wild bears

*Trichinella* larvae were detected in 7 of the 353 bears examined (2.0%, Table 1). The infection rate of brown bears in Hokkaido was 2.5%; at the subprefecture level, it was 18.2% in Sorachi, 5.3% in Shiribeshi, and 2.3% in Hiyama (Table 2). Among the 117 Japanese black bears examined, *Trichinella* larvae were detected in one bear (0.9%, Table 2) which was captured in Iwate Prefecture. All positive bears were adults, with LPG ranging from 0.3 to 180.0 (Table 3).

**Table 1:** Prevalence of *Trichinella* among the bears examined by species.

Host	Sex	No. of animals examined.	No. positive	Prevalence (%) (95% CI^b^)
Brown bear	Male	160	5	3.1 (1.1–7.3)
	Female	73	1	1.4 (0–8.1)
	N/A^a^	3	0	0 (0–79.8)
(Subtotal)		236	6	2.5 (1.0–5.6)
Japanese black bear	Male	77	0	0 (0–5.7)
	Female	29	1	3.4 (0–18.6)
	N/A^a^	11	0	0 (0–30.0)
(Subtotal)		117	1	0.9 (0–5.2)
Total		353	7	2.0 (0.9–4.1)

a N/A, not available.

b CI, confidence interval.

**Table 2:** Prevalence of *Trichinella* larvae in bears in Japan.

Locality			No. positive/No. examined	Prevalence (%) (95% CI^a^)
Prefecture	Region^b^	Subprefecture		
Hokkaido	Dohoku	Soya	0/4	0 (0–54.6)
		Kamikawa	0/7	0 (0–40.4)
	Doto	Okhotsk	0/3	0 (0–61.7)
		Nemuro	0/11	0 (0–30.0)
		Kushiro	0/3	0 (0–61.7)
		Tokachi	0/7	0 (0–40.4)
	Doo	Sorachi	2/11	18.2 (4.0–48.8)
		Ishikari	0/9	0 (0–34.5)
		Shiribeshi	2/38	5.3 (0.5–18.2)
		Iburi	0/2	0 (0–71.0)
		Hidaka	0/24	0 (0–16.3)
	Donan	Hiyama	2/87	2.3 (0.1–8.5)
		Oshima	0/30	0 (0–13.5)
	Subtotal		6/236	2.5 (1.0–5.6)
Aomori^c^			0/15	0 (0–23.9)
Akita			0/100	0 (0–4.4)
Iwate			1/2	50.0 (9.5–90.1)
	Subtotal		1/117	0.9 (0–5.2)
Grand total			7/353	2.0 (0.9–4.1)

a CI, confidence interval.

b Hokkaido is divided into four main regions: Dohoku (northern Hokkaido), Doto (eastern Hokkaido), Doo (central Hokkaido), and Donan (southern Hokkaido).

c Aomori, Akita, and Iwate are northern prefectures in Honshu Islands.

**Table 3:** Detailed information on *Trichinella*-infected bears and larvae per gram of *Trichinella.*

Host ID	Host	Localities	Location	Capture date	Sex	Age	LPG^a^
B1	Brown bear	Numata, Sorachi Subpref., Hokkaido	A	04-Jul-2020	Male	4	0.7
B2	Brown bear	Numata, Sorachi Subpref., Hokkaido	A	23-Jul-2020	Male	5	1.5
B3	Brown bear	Kaminokuni, Hiyama Subpref., Hokkaido	C	15-Sep-2020	Male	7	0.7
B5	Brown bear	Shimamaki, Shiribeshi Subpref., Hokkaido	B	27-Apr-2021	Male	12	0.3
B6	Brown bear	Kaminokuni, Hiyama Subpref., Hokkaido	C	19-Oct-2021	Male	8	4.0
B7	Brown bear	Shimamaki, Shiribeshi Subpref., Hokkaido	B	20-Oct-2021	Female	3–4	180.0
B4	Japanese black bear	Shiwa, Iwate Pref.	D	12-Dec-2020	Female	NE^b^	5.5

A–D: Location on the map (Fig. 1).

a LPG, larvae per gram.

b NE, not examined.

No significant differences in prevalence (P > 0.05) were observed between brown and Japanese black bears (Table 1). In terms of prevalence between sexes, that of males was 2.1% (n = 5/237, 95% CI: 0.8–5.0) and females 2.0% (n = 2/102, 95% CI: 0.1–7.3) with no significant difference. The sex of 14 of the bears was not recorded.

### 3.2 Genetic analysis of *Trichinella*

A partial sequence of the ITS2 region (362 bp) was determined for all positive samples. Multiple sequence alignment revealed that seven sequences were 100% identical. A BLAST search revealed 100% sequence identity (362/362 bp) with *Trichinella* T9 detected in the brown bear (accession no. AB255886). The top three other species (query coverage >95%) were *T. britovi* (<97.2%), *T. murrelli* (<96.1%), and *Trichinella* T6 (<95.2%).

A partial *cox1* sequence (345 bp) was determined from the same positive samples, and these sequences were 100% identical. BLAST analysis revealed 100% sequence identity to that of *Trichinella* T9 from Japanese black bears from Iwate Prefecture (accession no. LC546041) and brown bears from Hokkaido (accession no. LC361217) and 99.7% of the sequences from Japanese black bears in Iwate Prefecture (accession no. LC546040) and raccoons from Hokkaido (accession no. AB267879). Two haplotypes have been reported in Japanese black bears, tentatively named h1 and h2, for descriptive purposes. However, in this study, only h1 was detected from two bear species in Hokkaido and Iwate. The other species with query coverage of >95% was *T. murrelli* with <95.4%.

The representative sequences determined in this study have been deposited in the DNA Data Bank of Japan under accession nos. LC764456 (ITS2) and LC764591 (*cox1*).

## 4 Discussion

In this study, we identified the larvae detected in tongue muscle samples from six brown bears in Hokkaido and one Japanese black bear in Iwate as *Trichinella* T9, which is indigenous to Japan where the sylvatic cycle takes place. It has been reported that *Trichinella* T9 belongs to the same phylogenetic group as T6 and *T. nativa*; however, unlike them, T9 does not resist freezing (Pozio and Murrell, 2006). Notably, the *Trichinella* sequence registered as *T. britovi* from the Japanese black bear (accession no. AB091477) was 100% identical to the ITS2 sequence of *Trichinella* T9 obtained in this study (345/345 bp). Additionally, all other available sequences of *T. britovi* had <92.4% identity, implying that the sequence in the previous study was misregistered (Pozio and Murrell, 2006).

Two lineages of *Trichinella* T9 have been reported, which we tentatively named h1 and h2 haplotypes based on the differences in partial *cox1* sequences. Haplotype h1 was found in Japanese black bears in Iwate (accession no. LC546041, Tominaga et al., 2021) and brown bears in Hokkaido (accession no. LC361217, Tada et al., 2018), whereas haplotype h2 was detected in Japanese black bears in Iwate (accession no. LC546040, Tominaga et al., 2021) and raccoons from Hokkaido (accession no. AB267879, Kobayashi et al., 2011). In this study, neither h2 nor *T. nativa* was detected in bears in Hokkaido. Interestingly, a common haplotype—h1—was observed in both bear species in Iwate and Hokkaido, which are geographically separated by the Tsugaru Strait. This is the location of the Blakiston Line, a faunal and floral boundary between Hokkaido and Honshu Islands, Japan (Inoue et al., 2007). Clarifying the effect of the Blakiston Line on the genetic divergence of *Trichinella* T9 would have a significant meaning for biogeography.

Two species of bears exist in Japan: brown bears on the island of Hokkaido and Japanese black bears on the islands of Honshu and Shikoku, with their habitats separated by the Tsugaru Strait (Sato, 2010; Yamazaki, 2010). Both bear species are omnivores, feeding primarily on plant material, but occasionally hunt small animals or scavenge carcasses (Sato, 2010; Yamazaki, 2010; Matsubayashi et al., 2015), which transmit *Trichinella* in a predator-prey relationship, possibly regardless of their sex. The prevalence of *Trichinella* T9 in brown and Japanese black bears was 2.5% and 0.9%, respectively in this study. Given the low infection rates, wild bears in Japan may have limited contact with infected animals. Bears appear to be the dead-end hosts in the life cycle of *Trichinella*, as they are the highest-ranking species in natural ecosystems, especially in Japan. However, *Trichinella* larvae have a long lifespan in nurse cells within the muscle tissue, and they can survive for over 20 years in polar bears (Kumar et al., 1990). Since consuming bear carrion can transmit *Trichinella* to other animals, it has been suggested that wild bears play a role as long-term reservoirs of *Trichinella* in the natural environment. On the other hand, medium-sized carrion feeders, such as foxes, may be better suited as efficient sources of transmission in terms of the life cycle of *Trichinella*, probably including T9 (Campbell, 2018). In fact, red foxes had a relatively high prevalence of 13.8% (44/319, Kanai et al., 2007).

In recent years, the population and distribution of wild ungulates, particularly sika deer and wild boar, have increased throughout Japan. Furthermore, this trend has also affected wild bears, according to the Ministry of Agriculture, Forestry and Fisheries website (https://www.maff.go.jp/j/tokei/kouhyou/jibie/index.html). Consequently, the number of slaughtered and processed bears has almost doubled from 160 in the fiscal year 2016 to 306 in 2021 for human consumption and as food for other animals. The Guidelines for the Hygienic Management of Meat from Wild Animals in Japan state that wild game should not be eaten or served raw (Takai, 2022) because wild game, including bears, are prepared in slaughtering and processing facilities where veterinarians do not conduct inspections. Nevertheless, people still consume insufficiently cooked bear meat in restaurants, causing trichinellosis in Japan (Tada et al., 2018). *Trichinella* larvae were detected in wild bear meat in this study, albeit at low levels. Therefore, we believe sporadic trichinellosis outbreaks will likely continue if no further robust countermeasures are taken.

In conclusion, similar to the previous prevalence of 1.2–3.2% (Yamaguchi, 1991; Kanai et al., 2007), this study shows that *Trichinella* T9 is maintained at low levels in wild bears in the northern part of Japan (2.0%, 7/353). The findings imply that the recent human trichinellosis outbreaks, especially those that have occurred after 2016, might not be due to increased infection rates among wild bears, but due to increasing consumption and preference for wild game. To frame a clear and lucid answer to this matter, extensive surveys covering larger areas of Hokkaido and Honshu Islands would help obtain a comprehensive picture of *Trichinella* prevalence among wild bears and other wild animals. Owing to the growing popularity of wild meat dishes, the risk of *Trichinella* infection in the general population through undercooked or underprocessed bear meat consumption is increasing (Takai, 2022). Ongoing health education campaigns are highly necessary to raise awareness of the potential risk of trichinellosis among hunters, meat suppliers, consumers, and local government health agencies responsible for overseeing the public health of local populations.

## Funding

This study was financially supported partly by a grant from the 10.13039/501100003478Ministry of Health, Labour and Welfare, Japan (MHLW; 21KA1003, awarded to HS). The funders had no role in the study design, data collection, and interpretation, or in the decision to submit the work for publication.

## Declaration of competing interest

The authors declare that they have no conflicts of interest concerning this study.
